# Effectiveness of Sleep Deprivation in Treating Acute Bipolar Depression as Augmentation Strategy: A Systematic Review and Meta-Analysis

**DOI:** 10.3389/fpsyt.2020.00070

**Published:** 2020-02-25

**Authors:** Juan P. Ramirez-Mahaluf, Enzo Rozas-Serri, Fernando Ivanovic-Zuvic, Luis Risco, Paul A. Vöhringer

**Affiliations:** ^1^ Department of Psychiatry, Hospital Clínico, Facultad de Medicina, Universidad de Chile, Santiago, Chile; ^2^ Department of Psychiatry, School of Medicine, Pontificia Universidad Católica de Chile, Santiago, Chile; ^3^ Tufts Medical Center, Mood Disorders Program, Tufts University School of Medicine, Boston, MA, United States

**Keywords:** sleep deprivation, bipolar depression, meta-analysis, chronotherapy, mood medication, affective switch

## Abstract

**Background:**

Bipolar disorder is a disabling disease characterized by the recurrence of mood episodes. Successful strategies for the acute treatment of bipolar depression are still a matter of controversy. Total sleep deprivation (TSD) has shown acute antidepressant effect; however, the prompt relapse of depressive symptoms after sleep recovery has been reported. Taking this into consideration, we aimed to address a twofold research question: what are the acute effects of adding TSD to pharmacological treatment and what are the acute and chronic effects of adding medications to TSD.

**Methods:**

MEDLINE, Embase, Cochrane Central Register of Controlled Trials, and ClinicalTrials.gov databases were searched for clinical trials assessing bipolar depression and TSD. Two independent reviewers selected and classified 90 abstracts. The outcomes we assessed were change in Hamilton Depression Rating Scale (HDRS) or Montgomery–Asberg Depression Rating Scale (MADRS), sustained long-term response rate, treatment-emergent mania or hypomania, and tolerability (using dropout rates as a proxy). The compared groups were: TSD alone *versus* TSD plus medications and medications alone *versus* medications plus TSD. Data was analyzed using Stata 16.0.

**Results:**

Patients treated with TSD plus medications compared with medications alone showed a significant decrease in depressive symptomatology after one week (SMD −0.584 [95% CI −1.126 to −0.042], p = 0.03. Also, a significant decrease in depressive symptomatology (SMD −0.894 [95% CI −1.388 to −0.399], p < 0.001) was found in the group with TSD plus medications compared with TSD alone, at the 10^th^ day of treatment. We meta-analyzed the long-term effect of the TSD. It showed a sustained antidepressant effect (log OR = 2.365 (95% CI 0.95 to 3.779, p < 0.001) in the group where TSD was combined with medication when compared with patients treated only with TSD. Finally, no differences in tolerability (log OR = 0.234 (95% CI −1.164 to 1.632, p = 0.74) or affective switch were found.

**Conclusion:**

Adding TSD to medications to bipolar depression treatment resulted in an augmentation in acute response. We also found that medications have a positive impact in acute response when added to TSD. Furthermore, this higher response rate was maintained after 3 months while keeping Lithium therapy.

## Introduction

Bipolar disorder is a major health issue and cause of global disability (1). It is characterized by episodes of mania or hypomania and recurring depressive episodes that account for a considerable morbidity and mortality (2–4).

Depressive episodes, either in its major, dysthymic or mixed forms, are responsible for most of the morbidity in type I and type II bipolar patients at any stage of the disease progression and under any treatment (2, 4). Current treatments are more effective managing manic episodes than in depressive ones. The use of antidepressants reduces symptoms of acute bipolar depression, however, they do not increase clinical response or remission rates and have been associated with a long-term increased risk of treatment-emergent mania or hypomania (5). Cognitive impairment in bipolar depression is associated with dysfunction and disability and a higher rate of premature death when compared with the general population (6). This is caused by higher risk of suicide, accidents (6) and multiple medical causes (7). This highlights the need to explore effective treatment alternatives for acute bipolar depression.

Sleep deprivation (SD) is a treatment technique used for depression developed in the past 50 years (8, 9). Clinical studies have shown rapid antidepressive effects within 24–48 h after SD and even during the same night or following morning (10, 11). However, interest in their clinical use has diluted because it soon became evident that sleep recovery tended to reverse the clinical effect (12, 13). The best documented SD modality used with antidepressant purposes is Total sleep deprivation (TSD), though other modalities have been tested as shown in **Table 1** (14, 15).

**Table 1:** Sleep deprivation modalities.

Technique	Sleep Modality
**Total sleep deprivation (TSD)**	In one cycle of TSD patients are told to stay awake for about 36 h, from daytime until next day’s evening. Treatment usually consists in one to six cycles.
**Partial sleep deprivation (PSD)**	Sleep usually restricted to 4 to 5 h, either in the first (PSD-Late) or second half of the night (PSD-Early).
**Sleep phase advance (SPA)**	After one TSD cycle, starting with a scheduled time in bed from 17:00–24:00 h, with daily shift back of time in bed for 1 h, until original nocturnal sleep time is reached (23:00–06:00 h) after seven days. Usually intended to prevent relapse after effective TSD.
**Sleep phase delay (SPD)**	Time in bed from 02:00–07:00 h with a succeeding shift forward (30 minutes per day), until the initial sleep phase (23:00–06:00 h).

Historically, SD has shown response rates between 50 and 80% (12) in a wide spectrum of depressive disorders, with no differences by gender or age (13, 16). However, these rates are twice as high in so called endogenous depressions than in neurotic depressions or secondary ones (13, 16). Bipolar patients have even better outcomes when compared to endogenous recurrent unipolar ones (17). Other variables associated with SD response are: diurnal variation of mood (13, 14, 18), high level of thyroid function, low peripheral sympathetic activity, an altered Dexamethasone suppression test (11, 12), among others (19–23). Nevertheless, despite the mentioned and well established SD antidepressant effect, the clinical response to SD decreases rapidly (14). Relapse rates to SD are up to 83% after one night of sleep recovery (23); this has limited their clinical use. In order to avoid relapse, different SD schemes have been created, and the use of medication (serotonergic drugs, lithium salts) or other chronotherapeutic therapies has been added (12). Studies demonstrate that a combined treatment can lead to sustained euthymia after several months (12). However, the impact of the acute effect of SD combined with mood medications, as well as the long-term efficacy of TSD have only been evaluated in clinical studies with a limited number of patients. This makes it difficult to obtain conclusive results. In our search, only two previous meta-analysis about SD were found (18, 24), and none of them directly addressed the long-term effect of drug administration in combination with SD.

Therefore, we aimed to address a twofold research question: What are the acute effects of adding TSD to pharmacological treatment? and, What are the acute and long-term effects of adding medications to TSD?

## Methods

### Search Strategy and Selection Criteria

In this systematic review and meta-analysis, we searched MEDLINE, Embase, and the Cochrane Central Register of Controlled Trials databases from their inception until October 28, 2019, using the search term “(sleep AND deprivation AND bipolar AND depression)” with results filters based on study types (clinical trials and randomized controlled trials). We also searched ClinicalTrials.gov using the search term “(sleep AND deprivation AND bipolar AND depression)”. We reviewed references and citations of articles retained in this study for additional unidentified studies.

Inclusion criteria were: clinical trials recruiting patients aged 18–75 years undergoing a depressive episode with a primary diagnosis of bipolar I or bipolar II disorder according to the current versions of the DSM or ICD at the time of the respective trial. Patients must have received combined medication treatment and sleep deprivation therapy (with or without additional chronotherapy, see **Tables 2** and **3**). The existence of a control group was required.

**Table 2:** Characteristics of included trials in the first meta-analysis.

	Diagnosis	Medication (MED)	Chrono-therapy (CT)	Mean age (range or SD), years	Primary outcome instrument	Duration of treatment	MED			TSD+MED		
							n	D	AS	n	D	AS
Benedetti et al. (25) Nondouble-blind. Randomized controlled trial.	Bipolar depression (DSM-III-R)	Fluoxetine	TSD (3 cycles)	40 ± 12.1, 42 ± 9.7	HDRS-17	1 month	5	0	NR	5	0	NR
Wu et al. (11) Nondouble-blind. Randomized controlled trial.	Bipolar disorder, Major depressive episode (DSM-IV)	Lithium or other mood stabilizer, sertraline or other antidepressant	TSD (1 cycles), Bright light therapy, SPA (3 cycles)	39 ± 13.3, 40 ± 14.1	HDRS-19	7 weeks	17	0	0	32	5	2

MED, Medication; CT, Chronotherapy; SD, Standard deviation; DSM, Diagnostic and Statistical Manual; TSD, Total sleep deprivation; HDRS, Hamilton depression rating scale; SPA, Sleep phase advance; n, Sample size; D, Dropout; AS, Affective Switch.

**Table 3:** Characteristics of included trials in the second and third analysis.

	Diagnosis	Medication (MED)	Chrono-therapy (CT)	Mean age (range or SD), years	Primary outcome instrument	Duration of treatment	TSD			TSD+MED		
							n	D	AS	n	D	AS
Benedetti et al. (26) Nondouble-blind, Nonrandomized controlled trial.	Bipolar I (DSM-IV)	Lithium	TSD (3 cycles)	48.2 ± 10.8, 48.3 ± 11.2	HDRS- 21	3 months	20	0	NR	20	0	NR
Smeraldi et al (27) Double-blind, randomized controlled trial.	Bipolar I (DSM-IV) HDRS > 18	Pindolol	TSD (3 cycles) + Placebo	44.9 ± 11.5, 51.6 ± 12	HDRS- 21	6 months	20	0	0	20	0	0
Benedetti et al. (28) Nondouble-blind, Nonrandomized controlled trial.	Bipolar disorder, Major depressive episode (DSM-IV)	Lithium	TSD (3 cycles), SPA (3 cycles)	44.6 ± 8.9, 51.9 ± 11.6	HDRS-21	3 months	14	0	NR	16	0	NR
Benedetti et al. (29) Double-blind, randomized controlled trial.	Bipolar disorder, Major depressive episode (DSM-IV)	Amineptine	TSD (3 cycles) + Placebo	45 ± 10.9, 51.1 ± 10.3	MADRS	3 months	14	0	0	13	1	0
Colombo et al. (30) Nonrandomized controlled trial.	Bipolar disorder, Major depressive episode (DSM-IV)	Lithium	TSD (3 cycles) + Light exposure (intensity: 150–2500 lx)	45.4 ± 12.1, 46.4 ± 13.3	VAS	7 days	69	4	4	49	3	3

CT, Chronotherapy; MED, Medication; SD, Standard deviation; DSM, Diagnostic and statistical manual; TSD, Total sleep deprivation; HDRS, Hamilton depression rating scale; SPA, Sleep phase advance; n, Sample size; D, Dropout; AS, Affective Switch.

Exclusion criteria were: trials including inadequate samples (*i.e.* not exclusively bipolar patients or not control group) or interventions different from chronotherapeutic plus medication strategies. Studies with nonclinical outcomes (*i.e.* pharmacokinetics, serum concentrations, neuroimaging, *etc.*), or focused on clinical outcomes different from improvement, such as safety/tolerability, were also excluded. Studies where data could not be extractible or are not reported in raw form were excluded.

### Data Analysis

For each published study, summary estimates were extracted independently by JPRM and ERS, with subsequent verification (by PAV), review, and consensus. The following variables were extracted in a structured manner: patient characteristics (mean age, sex, and primary diagnosis), medication used (dose and titration schedule), chronotherapy technique used, and placebo condition, treatment-emergent hypomania or mania episodes, and dropouts.

The primary outcome was changed in clinician-rated depressive symptom score (Hamilton depression rating scale [HDRS] (31) or Montgomery–Åsberg Depression Rating Scale [MADRS] (32), calculated as standardized mean differences (SMDs) (33). The secondary outcome was clinical response at approximately 3 months, episodes of treatment-emergent mania or hypomania, and tolerability. We used odds ratios (ORs, with 95% CIs). Clinical remission was defined by the study’s authors as HDRS-21 score of 7 or less or MADRS score of 5 or less. We used dropout rates as a proxy for tolerability, although they might also reﬂect the absence of eﬃcacy.

Since treatment effects are likely to vary between studies due to methodological differences—such as patient selection, primary diagnosis, medication used, and trial duration—we used pooled random-effects models (34). Only intention-to-treat data was analyzed (35). Meta-regression procedures were developed to test the effect of time of treatment on SMDs.

In order to assess heterogeneity between trials (*i.e.* clinical and methodological diversity), Q statistics and I² were applied, with the threshold for heterogeneity defined as p value for the Q statistic of less than 0,1 or I² greater than 35% (36). Publication bias was evaluated by inspection of funnel plots and Egger’s test. Quality assessment was conducted with Cochrane Risk of Bias Tool for Randomized Controlled Trials (37). All analyses were performed using Stata 16.0 (StataCorp LP, College Station, TX, USA).

## Results

**Figure 1:**
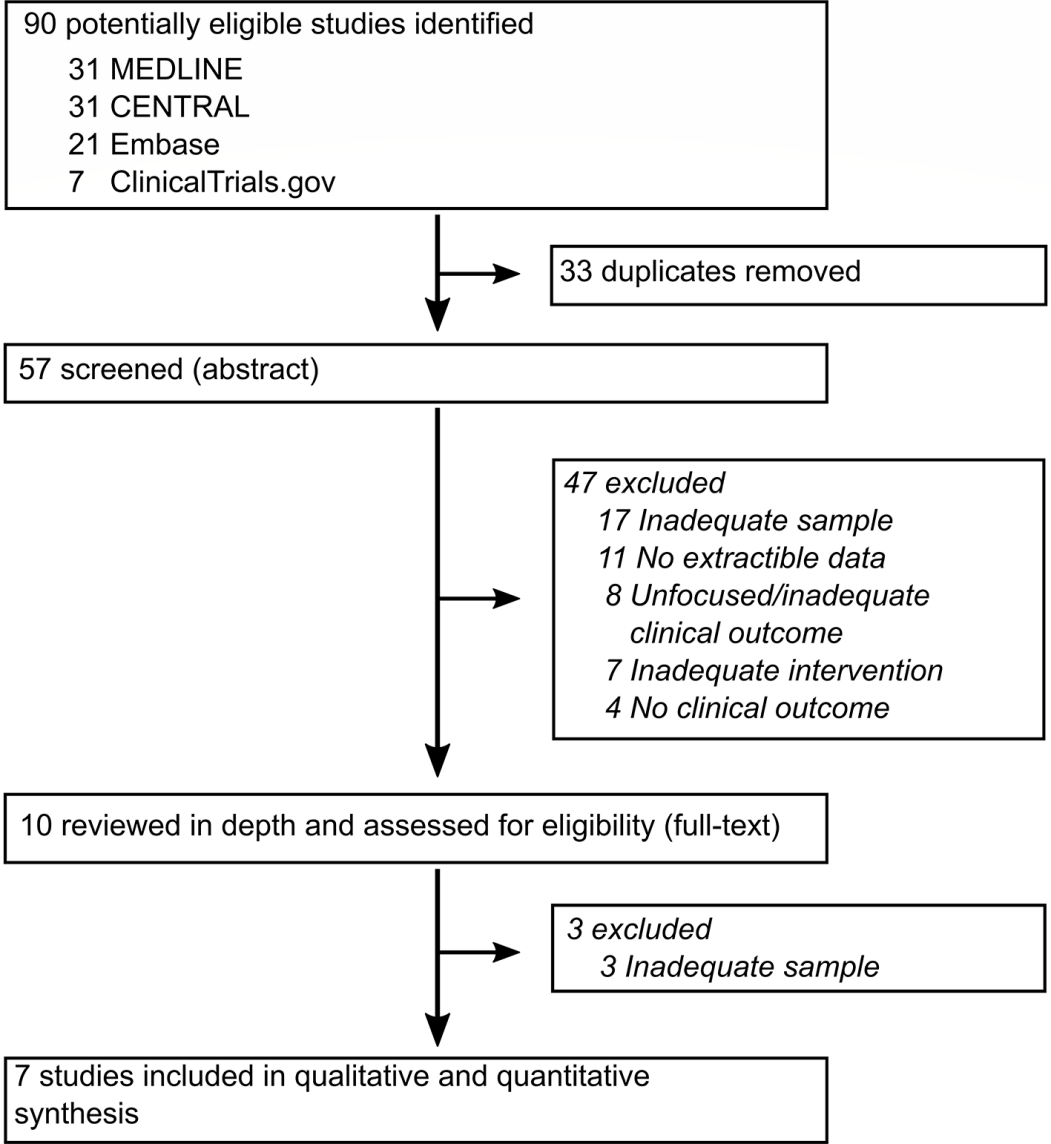
Study selection. CENTRAL, Cochrane Central Register of Controlled Trials.

We initially identified ninety potentially eligible studies (**Figure 1**). Removal of duplicates and screening of titles and abstract left ten records, of which three were excluded after full-text review because of inadequate mixed samples (38–40). One included study did not report clinical improvement on previously defined rating scales, however it reported dropout rates and episodes of treatment-emergent mania or hypomania (30).

Our meta-analysis included 311 bipolar patients with a depressive episode (80 with bipolar I disorder (DSM IV), 221 for whom the exact diagnosis was unspecified (DSM IV) and 10 with DSM-III-R bipolar disorder, **Tables 2** and **3**). Two trials included only type I bipolar patients. The mean age was 45.7 years (SD 4.2), and the overall sample consisted of 104 men and 170 women (the gender of 37 patients was not specified).

The seven selected trials (11, 25–30) included patients that used TSD as a chronotherapy. Of these seven, four used three cycles of TSD, one used three cycles of TSD plus light exposure (intensity of 150 or 2,500 lx), one used one cycle plus bright light and SPA, and one used three cycles of TSD plus three cycles of SPA (**Tables 2** and **3**). All seven trials used mood medications. In two studies (11, 25) both intervention and control groups received mood medication. There is no chance for a sleep deprivation placebo condition (**Table 2**). In the remaining five studies (26–30) both groups underwent TSD, only the intervention group received medication; of these five only two studies administered placebo to the control group (**Table 3**). The risk of bias assessment was conducted in all included studies, see supplementary material (**Figure S1** and **Table S1**).

### Clinical Effectiveness of TSD Addition to Mood Medication

**Figure 2:**
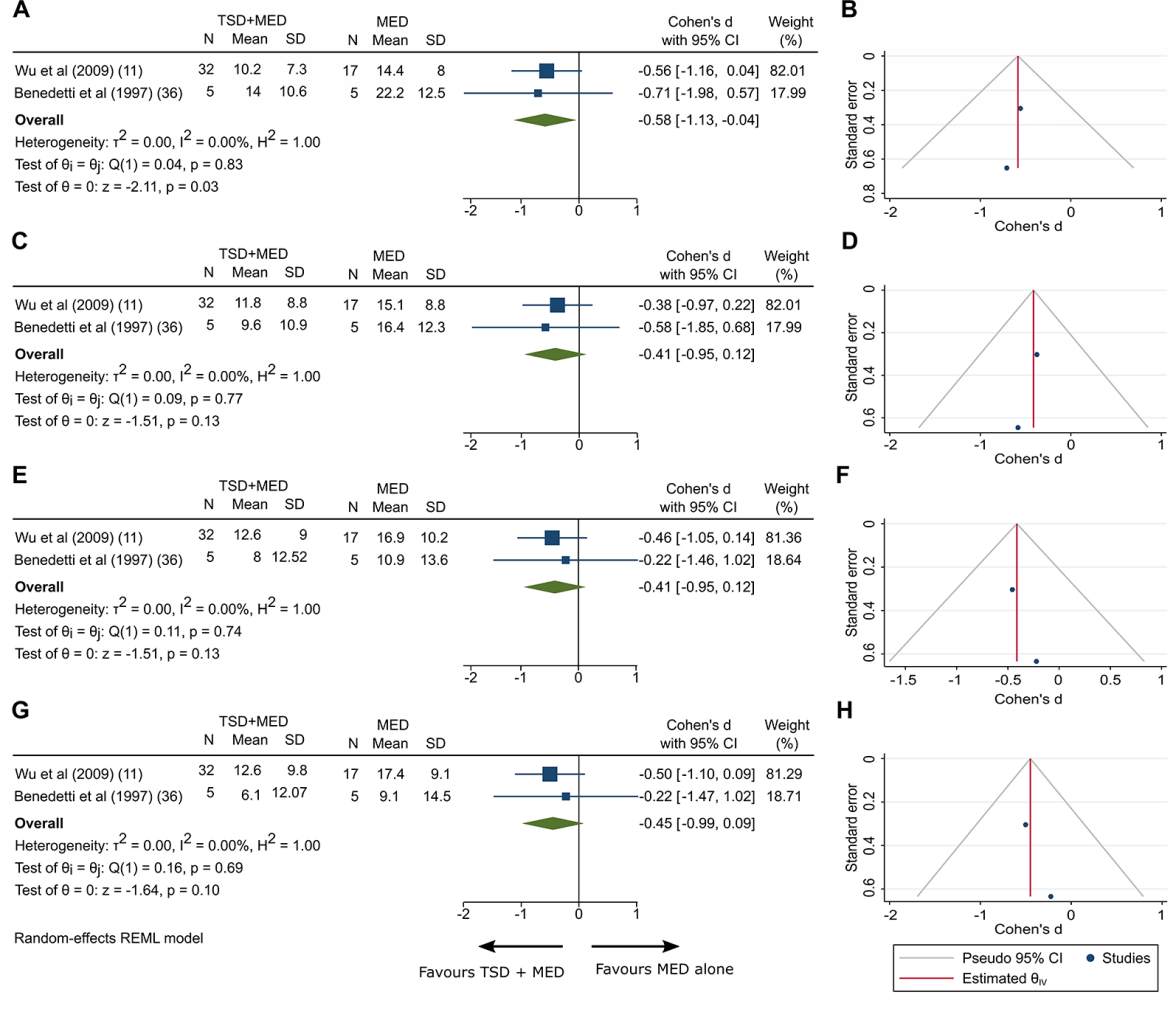
Clinician-rated depressive symptoms adding TSD to mood medication and the funnel plot of included studies at the 1^st^ week **(A**, **B)**, 2^nd^ week **(C**, **D)**, 3^rd^ week **(E**, **F)** and 4^th^ week **(G**, **H)**. TSD, Total sleep deprivation; MED, Medication; N; Sample size; CI, Confidence interval; SD, Standard deviation. Tests of heterogeneity, I^2^ and Q statistic were included. REML, Restricted maximum likelihood.

Depressive symptom scores were available for five trials, two of them comparing the addition of TSD to mood medication (11, 25). Symptoms were measured with HDRS-17 and HDRS-19 weekly. Both groups were treated with medications (fluoxetine and antidepressant/mood stabilizer, respectively), and one of the groups received additional three cycles of TSD and one cycle of TSD, bright light and SPA, respectively (**Table 2**). Compared with medication alone, adding TSD was associated with a significant improvement in clinician-rated depressive symptoms after 1 week (SMD −0.584 [95% CI −1.126 to −0.042], p = 0.03; **Figure 2A**). Between-sample heterogeneity was not significant, with a Q statistic of 0.04 (degree of freedom (df) = 1; p = 0.83) and a I^2^ of 0. Visual inspection of funnel plot showed asymmetry (**Figure 2B**).

The following weeks were associated with a nonsignificant tendency towards improvement in clinician-rated depressive symptoms: 2^nd^ week (SMD −0.413 [95% CI −0.949 to 0.124], p = 0.13; **Figure 2C**, for funnel plot see **Figure 2D**), 3^rd^ week (SMD −0.412 [95% CI −0.949 to 0.124], p = 0.13; **Figure 2E**, for funnel plot see **Figure 2F**), and 4^th^ week (SMD −0.45 [95% CI −0.988 to 0.088], p = 0.1; **Figure 2G**, for funnel plot see **Figure 2H**). To test the effect of time as a factor, we performed a meta-regression, that was nonsignificant (Coefficients 0.037 [95% CI −0.204 to 0.278], p = 0.765).

### Clinical Effectiveness of Medication Added to TSD at Day 10

**Figure 3:**
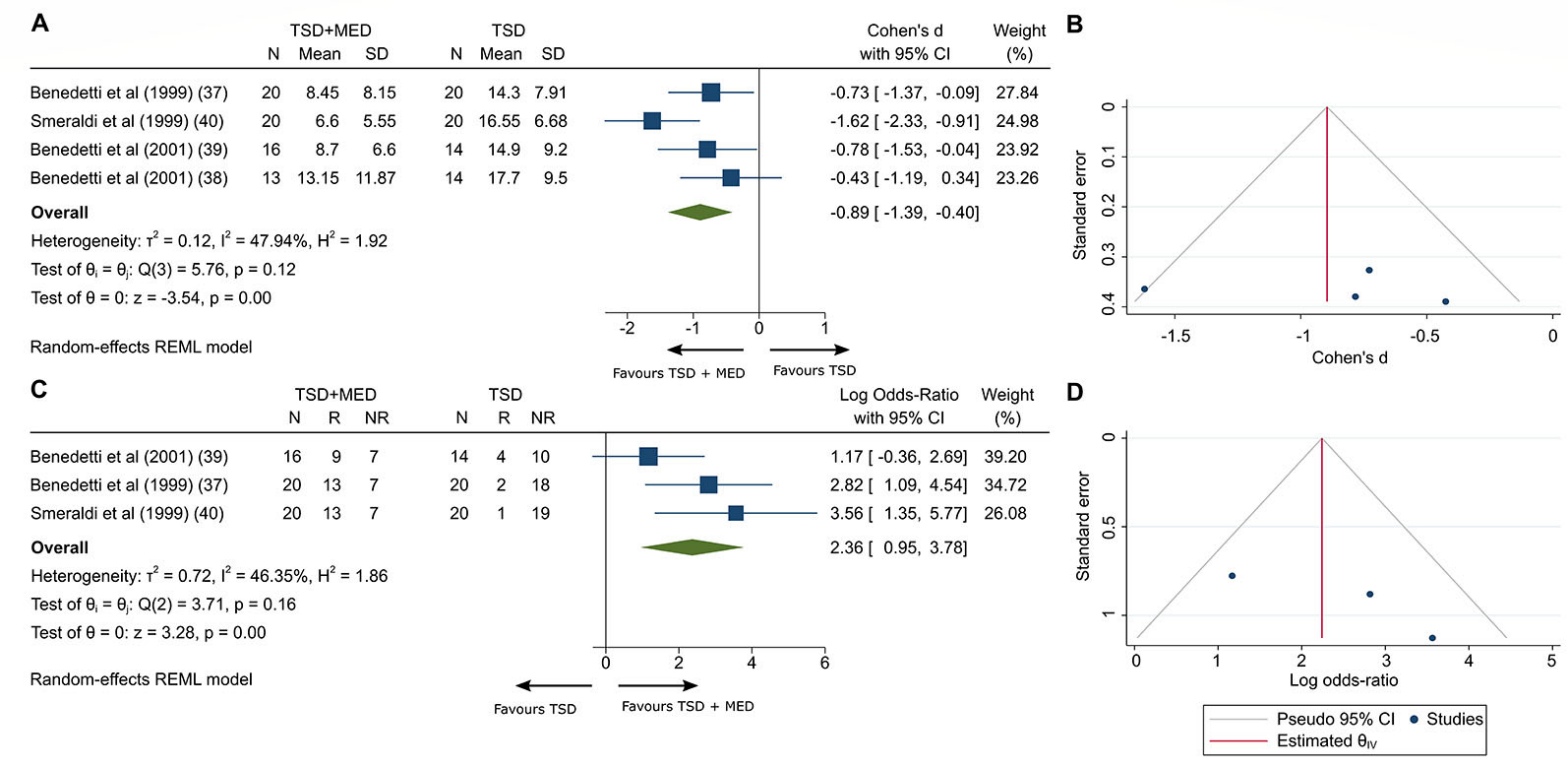
Clinician-rated depressive symptoms at the 10^th^ day adding medication to TSD **(A)** and the funnel plot of included studies **(B)**. Clinician remission at the 3^rd^ month **(C)** and the funnel plot of included studies **(D)**. TSD, Total sleep deprivation; MED, Medication; CI, Confidence interval; SD, Standard deviation; N, Sample size; R, Remission; NR, No remission; Tests of heterogeneity, I^2^ and Q statistic were included. REML, Restricted maximum likelihood.

Four trials compared the addition of mood medication to TSD (26–29). Depressive symptoms were measured with HDRS-21 and MADRS at the 10^th^ day. In three studies, patients were treated with three cycles of TSD, and in one of them patients were treated with three cycles of TSD and SPA. Mood medication (lithium, amineptine, and pindolol) was added to one group (**Table 3**). Compared with TSD alone, adding mood medication was associated with a significant improvement in clinician-rated depressive symptoms after 10 days of treatment (SMD −0.894 [95% CI −1.388 to −0.399], p < 0.001; **Figure 3A**). There was evidence of heterogeneity between the studies (I^2^ = 47.94, Q = 5.76, df = 3, p = 0.12). Visual inspection of funnel plot showed asymmetry (**Figure 3B**), but Egger test did not suggest small-study effects (*β*_1_ = 2.59, SE of *β*_1 =_ 12.8, z = 0.2, p = 0.84).

### Clinical Remission of Medication Added to TSD at 3 Months

Data for response rates were available for three trials that compared the addition of mood medication to TSD (26–28) (**Table 3**). Clinical remission was defined by the study authors as HDRS-21 score < 8. Pooled log OR was 2.365 (95% CI 0.95 to 3.779, p < 0.001; **Figure 3C**), compared with TSD, adding mood medication was associated with a significant clinical remission after 3 months. There was evidence of heterogeneity between the studies (I^2^ = 46.35, Q = 3.71, df = 2, p = 0.16). Visual inspection of funnel plot showed slight asymmetry (**Figure 3D**), however Egger test did not suggest small-study effects (*β*_1_ = 6.71, SE of *β*_1_ = 3.9, z = 1.72, p = 0.0854).

### Tolerability and Treatment-Emergent Mania or Hypomania

The rates of treatment-emergent mania or hypomania were available for four studies (11, 27, 29, 30). At the end of the treatment, five (4.35%) of 115 patients given medication combined with TSD, four (4.17%) of 96 patients given TSD and zero (0%) of 17 patients given mood medication had episodes of treatment-emergent mania or hypomania. Due to different control groups in the studies that reported aﬀective switch (11, 30), it was not possible to pool the data in a meta-analysis (**Tables 2** and **3**).

**Figure 4:**
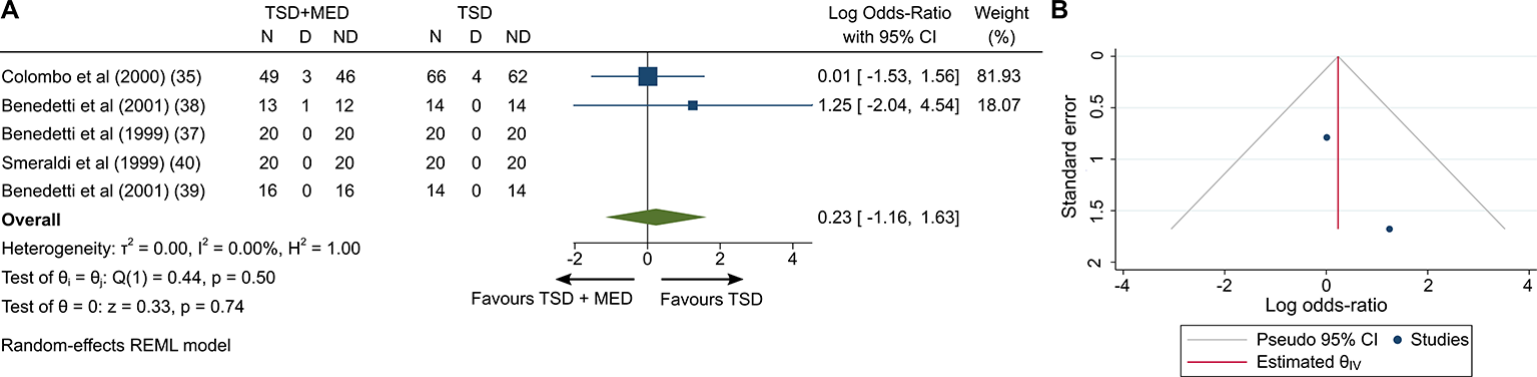
Tolerability at the end of the trial adding medication to TSD **(A)** and the funnel plot of included studies **(B)**. TSD, Total sleep deprivation; MED, Medication; CI, Confidence interval; SD, Standard deviation; N, Sample size; D, Dropout; NR, No dropout; Tests of heterogeneity, I^2^ and Q statistic were included; REML, Restricted maximum likelihood.

The rates of dropout were available for all studies (11, 26–30). Overall, nine (5.8%) of 155 patients given mood medication combined with TSD, four (2.92%) of 137 given TSD and zero (0%) of 17 given mood medication were lost to follow-up (**Tables 2** and **3**). Pooled log OR was 0.234 (95% CI −1.164 to 1.632, p = 0.74; **Figure 4A**) compared with TSD; adding mood medication was not associated with differences tolerability (dropout rate). Between-sample heterogeneity was not significant (I^2^ = 0, Q = 0.44, df = 1, p = 0.5). Visual inspection of funnel plot showed slight asymmetry (**Figure 4B**).

## Discussion

This meta-analysis provides three main results regarding the efficacy of Sleep deprivation (SD) in bipolar depression. Our first result suggests that adding TSD to medication acutely increases the antidepressant effectiveness relative to mood medications alone at the first week, although this effect was not maintained in the following weeks. The second result shows that the addition of medication to TSD increased antidepressant effectiveness at day 10 of treatment relative to TSD alone. The third result yields the conclusion that the use of medication plus TSD increased clinical remission at a 3-month follow-up. Finally, regarding tolerability and treatment-emergent mania or hypomania, our results show no differences between treatments.

We initially proposed a twofold research question. These results suggest that chronotherapeutic strategies such as SD, regardless of their long-term efficacy, may be useful therapeutic tools to treat the symptoms of bipolar depression while the initial effect of antidepressant drugs is achieved. This finding is consistent with the widely documented rapid-onset antidepressant effects of SD (10, 14, 23). It should be considered that, even though drugs’ antidepressant effects increase over a period of weeks, the impact of chronotherapeutic strategies was not statistically significant at week 2 or further on. SD’s use in the medium or long term would not be so well justified if pharmacological therapy is already effective. Furthermore, if mood medication shows a synergistic effect on short-term follow-up, there is no reason to use TSD without drugs. A possible exception would be patients who have an absolute contraindication for mood medication use. Relative to the long-term effects of adding medication to TSD, such strategy suggests a much lower rate of symptomatic relapse after recovery from sleep when TSD is used alone. Our results showed that concomitant use of mood medications and TSD maintained clinical remission on the long-term follow-up phase.

In our search, only two previous meta-analysis related to SD were found. The first (18) studied diurnal variation as a response predictor for SD. Recently another meta-analysis was published including a total of 66 studies with a huge heterogeneity (different SD modalities, different depressive samples, demographics and different definitions of treatment response) and showed an overall response rate of 45% among the six randomized studies and 50% among the remaining nonrandomized included studies (24). Our results differed from this meta-analysis since they did not find a significant effect of adding medications to SD. The fact that they included unipolar, bipolar, and mixed patient samples, and did not make a difference between short- and long-term outcomes might explain our different results. Moreover, the same meta-analysis concluded that response rate was not significantly affected by SD modality or sample type (unipolar, bipolar, or mixed). Unlike what has been reported in previous publications, the results of that meta-analysis indicate a nonsignificant tendency of a lower response in bipolar patients when compared to unipolar patients. This might be explained by the exclusion of studies in which other chronotherapeutic strategies were applied in conjunction with SD that show better response rates. For this reason, the authors propose that bipolar patients could benefit from SD mainly when it is associated with other chronotherapeutic therapies.

Recent clinical guidelines in Major Depression Disorder (41), consider SD as a third-line adjunctive therapy for more severe and refractory forms of MDD in combination with other chronotherapeutic strategies. On the other hand, bipolar disorder clinical guidelines (42), consider chronotherapeutic strategies as novel or experimental options and are therefore seen as the last step among therapeutic alternatives. A recent systematic review claim that sleep deprivation research provides a low level of evidentiary support, mainly from uncontrolled studies, for the acute management of bipolar depression (43). Additionally, some promoters of TSD therapies propose its use even as a first-line strategy for people affected by mood disorders, given its safety (14). On the basis of our results, patients treated with TSD, with the concomitant use of mood medications (Lithium and monoaminergic drugs were used in the included trials) resulted in a rapid and sustained long-term antidepressant effect. TSD seems to be a recommended strategy in bipolar patients who fall into a depressive episode while keeping the previous treatment with mood medications such as lithium.

This study has some limitations and entails further research questions and challenges. The first and probably major limitation is related to the small number of clinical trials available in literature that could meet inclusion criteria. It is important to note that, although there are many studies with available data about response rates and even relapse rates in patients subjected to different modalities of sleep deprivation, many of these have different methodologies and objectives than those analyzed in our study, such as functional brain studies or analysis of biological markers in responder patients versus non-responders to sleep deprivation. However, for the purposes of our initial research questions, clinical trials that directly assessed the impact of pharmacological and chronotherapeutic interventions in comparable terms were necessary. This research feature limited the number of studies that meet the inclusion criteria and showed that this is a research area in which new clinical trials are strongly needed to obtain more conclusive results. This would allow for more informed clinical decision making. Another limitation of this study is related to the small number of patients that each selected clinical trial recruited. The very difficulties that come when implementing these treatment modalities could explain low participant numbers. Recruiting volunteers or patients willing to receive these treatments can be challenging. Moreover, the included trials had differences in dependent and independent variables, such as: differences in prescribed mood medications (different antidepressants and Lithium) and doses, outcome scales (VAS, HDRS, MADRS), and chronotherapeutic modalities (PSD, TSD with or without light therapy or other biological treatments), placebo conditions and diagnosis criteria. These differences must be considered as limitations for the generalization of our findings.

Although a new timeframe criterion for hypomania was added in DSM IV compared to DSM-III-R (44) (the two versions used in the trials included in this meta-analysis), this is probably a comparatively minor limiting factor. However, the evolution of the definition of bipolar disorder type I, type II, and bipolar spectrum disorders is an issue to be considered in the heterogeneity of the samples in future research.

These limitations could not only significantly restrict the generalization of our findings, but they are also a call for researchers to consider lowering levels of heterogeneity in key methodological parameters such as randomization and placebo medication, despite the inherent difficulties of chronotherapy research. Furthermore, trials have not yet systematically addressed other important questions such as the differential effect of TSD regarding bipolar subtypes.

## Conclusion

Our results suggest that adding chronotherapeutic interventions to mood medications for depressive episodes in bipolar patients seems to increase the antidepressant effect of the drugs early on of the treatment and maintaining the mood effects for the long-term follow-up.

## Author Contributions

JR-M, ER-S, PV, and LR conceived the study. JR-M and ER-S did the literature review, statistical analyses, and drafted the report. JR-M and ER-S extracted the data. All authors interpreted the data and edited and approved the ﬁnal report.

## Funding

Work by JR-M was funded by CONICYT PIA ACT 1414 and CONICYT FONDECYT *(Ref:* 3190311). PV has received salary support through the Fund for Innovation and Competitiveness (FIC) of the Chilean Ministry of Economy, Development and Tourism, through the Millennium Scientiﬁc Initiative (grant number IS130005). The cost of publication was funded by Fundacion Hospital Clínico de la Universidad de Chile.

## Conflict of Interest

The authors declare that the research was conducted in the absence of any commercial or financial relationships that could be construed as a potential conflict of interest.
