# Supplementary material for: Effectiveness of Sleep Deprivation in Treating Acute Bipolar Depression as Augmentation Strategy: A Systematic Review and Meta-Analysis
Source: Front Psychiatry. 2020 Feb 25;11:70. doi: 10.3389/fpsyt.2020.00070 (PMC7052359; doi:10.3389/fpsyt.2020.00070)
Supplement: Supplementary file 1 [file DataSheet_1.pdf]

Supplementary Material for

Effectiveness of Sleep Deprivation in Treating Acute Bipolar Depression as Augmentation Strategy: A Systematic Review and Meta-Analysis

Juan P. Ramírez-Mahaluf, Enzo Rozas-Serri, Fernando Ivanovic-Zuvic, Luis Risco, Paul A. Vöhringer

This PDF file includes:

- **Figure S1**
- **Table S1**

Quality assessment of included studies

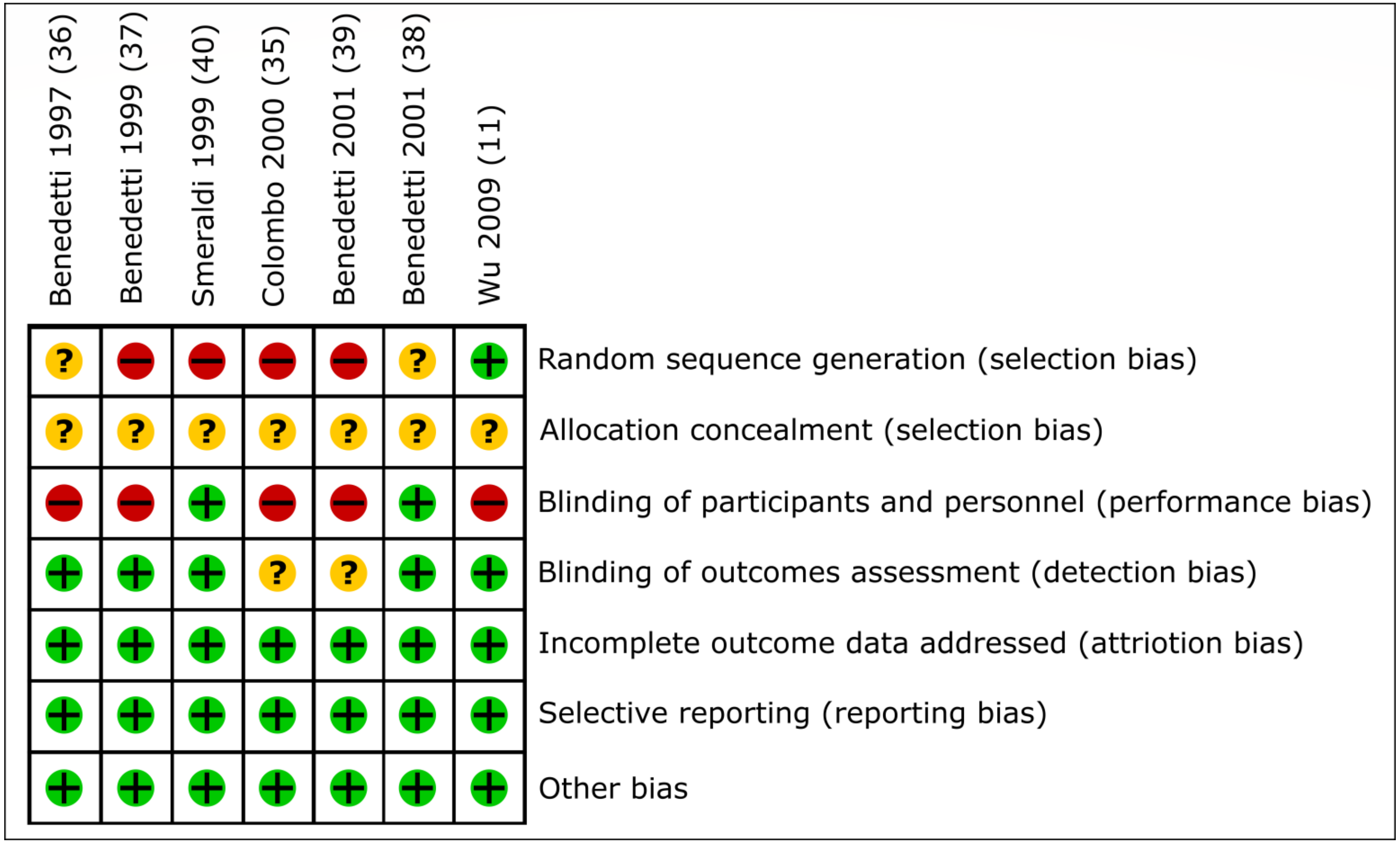

Fig S1. Assessment of risk of bias of clinical trials: Red: High risk, Yellow: Unclear risk, Green: Low risk. Details about the reasons for this assessment are listed in Table S2.

Table S2. Assessment of risk of bias of clinical trials with reasons.

Benedetti 1997 (36)

**Bias**	**Judgement**	**Support for judgement**
Random sequence generation (selection bias)	Unclear risk.	Quote: "At the outset of the study, patients were enrolled and randomly assigned to the two treatment groups"<br>Comment: Randomization method not mentioned.
Allocation concealment (selection bias)	Unclear risk.	Comment: Not mentioned in the article.
Blinding of participants and personnel (performance bias)	High risk.	Comment: The allocated intervention is unable to be blinded in this study.
Blinding of outcome assessment (detection bias)	Low risk.	Quote: "Severity of depression and mood change were assessed on the mornings of the outset, days 7, 14, 21, and 28 by external raters who were blind to the experimental conditions".<br><br>Comment: Probably done.
Incomplete outcome data addressed (attrition bias)	Low risk.	Comment: No missing outcome data.
Selective reporting (reporting bias)	Low risk.	Comment: The study protocol is available and outcomes have been reported in the pre-specified way.
Other bias	Low risk.	Comment: The study appears to be free of other sources of bias.

Benedetti, 1999 (37)

**Bias**	**Judgement**	**Support for judgement**
Random sequence generation (selection bias)	High risk.	Quote: “Patients were assigned to two groups on the basis of the presence (group 1, N = 20) or absence (group 2, N = 20) of long term lithium treatment”<br>Comment: Non-random categorization of participants, allocation by availability of the intervention.
Allocation concealment (selection bias)	Unclear risk.	Comment: Not mentioned in the article.
Blinding of participants and personnel (performance bias)	High risk.	Comment: No blinding of the allocated intervention.
Blinding of outcome assessment (detection bias)	Low risk.	Quote: “Objective mood ratings were obtained by administering the 21-item HAM-D at day 0 and at day 10 (i.e., 4 days after the last TSD cycle). Whenever possible, the same rater (blind to the treatment option) conducted admission and follow-up ratings for each patient.”<br>Comment: Probably done.
Incomplete outcome data addressed (attrition bias)	Low risk.	No missing data.
Selective reporting (reporting bias)	Low risk.	Comment: The study protocol is available and outcomes have been reported in the pre-specified way.
Other bias	Low risk.	Comment: The study appears to be free of other sources of bias.

Smeraldi 1999 (40)

**Bias**	**Judgement**	**Support for judgement**
Random sequence generation (selection bias)	Low risk.	Quote: "Patients were randomly assigned to two treatment groups (day 0): TSD plus pindolol (Group 1, *n*=20) or TSD plus placebo (Group 2, *n*=20); randomization was performed by a computer generated schedule".<br>Comment: Probably done.
Allocation concealment (selection bias)	Unclear risk.	Comment: Not mentioned in the article.
Blinding of participants and personnel (performance bias)	Low risk.	Quote: "From day 1 to day 9 patients were double-blind treated TID with pindolol 2.5 mg or placebo".<br>Comment: Probably done.
Blinding of outcome assessment (detection bias)	Low risk.	Quote: "The 21-item HDRS was administered by trained raters who were blind to treatment option at day 0 and at day 10".<br>Comment: Probably done.
Incomplete outcome data addressed (attrition bias)	Low risk.	No missing data.
Selective reporting (reporting bias)	Low risk.	Comment: The study protocol is available, and outcomes have been reported in the pre-specified way.
Other bias	Low risk.	Comment: The study appears to be free of other sources of bias.

Colombo 2000 (35)

Bias	Judgement	Support for judgement
Random sequence generation (selection bias)	High risk.	Quote: "Forty-nine had been taking lithium salts for at least 6 months before the onset of the current depressive episode, while 66 had not been placed on a long term medication by previous psychiatrists in charge."<br>Comment: Non-random categorization of participants, allocation by availability of the intervention.
Allocation concealment (selection bias)	Unclear risk.	Comment: Not mentioned in the article.
Blinding of participants and personnel (performance bias)	High risk.	Comment: No blinding of the allocated intervention.
Blinding of outcome assessment (detection bias)	Unclear risk.	Comment: Not mentioned in the article.
Incomplete outcome data addressed (attrition bias)	Low risk.	"Seven patients switched polarity during the TSD treatment and were excluded from further study. Three of these had been taking lithium salts and were treated with red 150 lux light, and four were without lithium and were treated with red (*n*=2) and bright white light (*n*=2).<br>Comment: Reasons for missing outcome data unlikely to be related to true outcome.
Selective reporting (reporting bias)	Low risk.	Comment: The study protocol is available, and outcomes have been reported in the pre-specified way.
Other bias	Low risk.	Comment: The study appears to be free of other sources of bias.

Benedetti 2001 (39)

Bias	Judgement	Support for judgement
Random sequence generation (selection bias)	High risk.	Quote: “Patients were assigned to two groups based on the presence (Group 1, n=16) or absence (Group 2, n=14) of long-term lithium treatment.”<br>Comment: Non-random categorization of participants, allocation by availability of the intervention.
Allocation concealment (selection bias)	Unclear risk.	Comment: Not mentioned in the article.
Blinding of participants and personnel (performance bias)	High risk.	Comment: No blinding of the allocated intervention.
Blinding of outcome assessment (detection bias)	Unclear risk.	Quote: “Objective mood ratings were obtained every day”.<br>Comment: Not mentioned in the article.
Incomplete outcome data addressed (attrition bias)	Low risk.	No missing data.
Selective reporting (reporting bias)	Low risk.	Comment: The study protocol is available, and outcomes have been reported in the pre-specified way.
Other bias	Low risk.	Comment: The study appears to be free of other sources of bias.

Benedetti 2001 (38)

**Bias**	**Judgement**	**Support for judgement**
Random sequence generation (selection bias)	Unclear risk.	Quote: “On day 0, patients were enrolled and randomly assigned to two treatment groups: amineptine, 200 mg o.d. (group 1, n = 14) or placebo (group 2, n = 14)”.<br>Comment: Randomization method not mentioned.
Allocation concealment (selection bias)	Unclear risk.	Not mentioned in the article.
Blinding of participants and personnel (performance bias)	Low risk.	Quote: “Treatments were administered in a double-blind design from day 1 to day 9”.<br>Comment: Probably done.
Blinding of outcome assessment (detection bias)	Low risk.	Quote: “by trained raters blind to treatment options on days 1, 2 and 7”.<br>Comment: Probably done.
Incomplete outcome data addressed (attrition bias)	Low risk.	No missing data.
Selective reporting (reporting bias)	Low risk.	Comment: The study protocol is available, and outcomes have been reported in the pre-specified way.
Other bias	Low risk.	Comment: The study appears to be free of other sources of bias.

Wu 2009 (11)

Bias	Judgement	Support for judgement
Random sequence generation (selection bias)	Low risk.	Quote: "Following initial screening, patients were randomly assigned (JCW) using a rand om number generator program to a chronotherapeutic augmentation treatment (CAT) gro up (on a 3:2 ratio for follow-up studies; n=32) or a medication-only (MED) group (n=1 7)."<br>Comment: Probably done.
Allocation concealment (selecti on bias)	Unclear risk.	Comment: Not mentioned in the article.
Blinding of participants and p ersonnel (performance bias)	High risk.	Quote: "It was not possible to blind patients or raters to SD procedure"<br>Comment: The allocated intervention is unable to be blinded in this study.
Blinding of outcome assessme nt (detection bias)	Low risk.	Quote: "It was not possible to blind patients or raters to SD procedure; interviews were videotaped to assess and maintain interrater reliability"<br><br>Comment: No blinding of outcome assessment, but the review authors judge that the ou tcome measurement is not likely to be influenced by lack of blinding
Incomplete outcome data addr essed (attrition bias)	Low risk.	Quote: "During follow-up, five patients in the CAT group terminated early because of rel ocation (n=1), intolerance to medications (n=2), or failure to adhere to protocol during fo llow-up (n=2). None in the MED group terminated early.

		Comment: Reasons for missing outcome data unlikely to be related to true outcome.
Selective reporting (reporting bias)	Low risk.	Comment: The study protocol is available and outcomes have been reported in the pre-specified way.
Other bias	Low risk.	Comment: The study appears to be free of other sources of bias.
